# Hernia of the Ovary Mistaken for an Encysted Tumour—Extirpation—Death

**Published:** 1851-11

**Authors:** 


### OBSTETRICS.

*Hernia of the Ovary mistaken for an Encysted Tumour—Extirpation—Death.* By M. EVERSANT.—A girl, eleven years of age, came into the hospital to be under the care of M. Eversant, having a tumor in the left labium. Her parents had observed it when the girl was a year old, but it had never caused any trouble till eighteen months previously, when it began to be painful, and made the patient walk lame. The tumor was very moveable, more moveable than a cyst. It was easily pushed from the inguinal ring to the lowest part of the labium—like a testicle in the scrotum. It was of the size of a small nut, painful on pressure, and in one point fluctuation could be perceived.

It was diagnosed to be an encysted tumor, and it was resolved to extirpate it. An incision was made in the centre of the labium, and the dissection carefully continued layer by layer. A cord similar to the spermatic cord, and containing part of the fallopian tube, was arrived at and tied. It was then cut across below the ligature, and the ovary removed along with the cord attached.

The girl shortly took peritonitis, and died on the third day after the operation.—*London Monthly Journal from Gazette de Hôpitaux*, 24th May 1851.

